# LL-37 causes cell death of human nasal epithelial cells, which is inhibited with a synthetic glycosaminoglycan

**DOI:** 10.1371/journal.pone.0183542

**Published:** 2017-08-24

**Authors:** Andrew J. Thomas, Abigail Pulsipher, Brock M. Davis, Jeremiah A. Alt

**Affiliations:** 1 Division of Head and Neck Surgery, Rhinology - Sinus and Skull Base Surgery Program, Department of Surgery, University of Utah School of Medicine, Salt Lake City, Utah, United States of America; 2 GlycoMira Therapeutics, Salt Lake City, Utah, United States of America; Hospital of the University of Pennsylvania, UNITED STATES

## Abstract

LL-37 is an immune peptide that regulates innate and adaptive immune responses in the upper airways. Elevated levels of LL-37 have been linked to cell death and inflammatory diseases, such as chronic rhinosinusitis (CRS). Glycosaminoglycans (GAGs) are polysaccharides that are found on respiratory epithelial cells and serve important roles in mucosal surface repair. Recent findings suggest that a synthetic glycosaminoglycan (GM-0111) can protect against LL-37-induced sinonasal mucosal inflammation and cell death in a murine model of acute RS. Herein, we elucidated the mechanisms by which LL-37 causes sinonasal inflammation and how GM-0111 can prevent these mechanisms. When challenged with LL-37, human nasal epithelial cells (HNEpCs) and mouse macrophages (J774.2) demonstrated increased release of adenosine triphosphate (ATP) and interleukin (IL)-6 and -8, as well as cell death and lysis. These cellular responses were all blocked dose-dependently by pre-treatment with GM-0111. We identified that LL-37-induced cell death is associated with caspase-1 and -8 activation, but not activation of caspase-3/7. These responses were again blocked by GM-0111. Our data suggest that LL-37 causes cellular death of HNEpCs and macrophages through the pro-inflammatory necrotic and/or pyroptotic pathways rather than apoptosis, and that a GM-0111 is capable of inhibiting these pro-inflammatory cellular events.

## Introduction

Chronic rhinosinusitis (CRS) is a debilitating condition of sinonasal mucosal inflammation that affects up to 49 million Americans.[1,2,3,4,5] Patients with CRS experience significant declines in quality of life more disabling than other chronic conditions such as coronary heart disease and Parkinson’s Disease.[6,7,8,9,10,11] Despite its large impact on society, the pathogenesis of this condition remains unclear, as CRS is complex with multiple etiologies (*e*.*g*., allergen, bacterial, viral, or fungal exposure).[12,13,14,15] Regardless of etiology, a seemingly unchecked state of persistent inflammation is common in most patients. Immunological hallmarks of CRS include increased permeability and damage to the sinonasal epithelial cell barrier induced by epithelial cell death and the infiltration of innate and adaptive immune cells. Current treatments of CRS primarily target nasal symptom relief and sinonasal inflammation with intranasal steroid sprays.[4] However, approximately 20% of patients are unresponsive to available medical therapies and turn to endoscopic sinus surgery as an alternative,[2,16,17] underscoring the need for improved anti-inflammatory therapeutics.

We previously developed a murine model of sinonasal mucosal inflammation to mirror the underlying inflammatory state of rhinosinusitis through intranasal instillation of the inflammatory-modulating peptide LL-37. This model was also developed for testing potential anti-inflammatory therapeutics capable of preventing and reducing such inflammatory effects. LL-37 has cytotoxic effects on bronchial and alveolar epithelial cells and has been shown to enhance mucus production in the airways, contributing to the pathogenesis of chronic obstructive pulmonary disorder.[18,19] LL-37 is expressed in many different cell types, including upper airway epithelial cells, immune cells, and cells comprising the sinonasal mucosa [20,21,22,23] and is upregulated in the nasal secretions and sinonasal mucosa of CRS patients.[24,25] In this model, we found robust responses consistent with RS-related changes, including histologic evidence of inflammation of the mucosa and increased inflammatory cell infiltration and cell death.[26] We further reported that a synthetic glycosaminoglycan (GAG; GM-0111) can prevent these inflammatory changes by blocking cell death and immune cell infiltration into the sinonasal epithelium and mucosa.[27] GAGs are polysaccharides normally found on respiratory epithelial cells and mucosal glands which are known to play important and diverse roles in the upper airways, including fluid homeostasis, inflammation, and mucosal tissue repair and remodeling.[28,29,30,31,32]

In the present study, we elucidated the molecular mechanisms through which LL-37 might be inducing sinonasal mucosal inflammation and how GM-0111 can block these mechanisms. The specific death pathways being activated and inhibited were examined on the single cell level using human primary nasal epithelial cells (HNEpCs) and mouse monocyte macrophage cell line J744.2 as an *in vitro* model of sinonasal mucosal inflammation. Using this model, secreted factors indicative of cellular stress (adenosine triphosphate (ATP)) and cytotoxicity (interleukin (IL)-6 and IL-8) were quantitated, whereas cell morphological changes were qualitatively interpreted within the context of sinonasal mucosal inflammation.

## Materials and methods

### Reagents

LL-37 is a C-terminal peptide fragment from human cathelicidin with a sequence of LLGDFFRKSKEKIGKEFKRIVQRIKDFLRNLVPRTES. LL-37 was obtained from the DNA/Peptide Synthesis Core Facility at the University of Utah (Salt Lake City, UT) at >95% purity. GM-0111 was supplied by GlycoMira Therapeutics (Salt Lake City, UT).[33] Compounds were dissolved in NanoPure double-distilled water (ddH_2_O) or phosphate buffered saline (PBS; pH 7.4) and filtered through a sterile 0.22 μm filter before use.

### Cell culture

HNEpCs and recommended cell culture supplies were obtained from Celprogen (Torrance, CA). J774.2 cells, a BALB/C mouse monocyte macrophage cell line, were obtained from Sigma Aldrich (St. Louis, MO); the recommended cell culture supplies for J774.2 cells were obtained from ThermoFisher Scientific (Grand Island, NY). Cells were maintained at 37°C and 5% CO_2_. All expanding, freezing, and culturing protocols were performed according to the suppliers’ instructions.

### ATP release and death quantitation of HNEpCs and J774.2 cells

For analyses of LL-37-induced ATP release and cell death, HNEpCs and J774.2 cells were first detached from culture flasks using Accutase (Innovative Cell Technologies; San Diego, CA), delivered to complete medium, pelleted by centrifugation, and then resuspended in 1 mL of complete medium. Cells were counted using a hemocytometer, examined for viability with trypan blue (0.4% solution, Thermo Fisher Scientific; Hampton, NH), and only used when the population was >90% viable. For ATP, cell death, and caspase assays the HNEpCs and J774.2 cells were plated into 24-well plates at a density of 500,000 cells/well. For ELISA assays HNEpCs were plated in 96-well plates at a density of 10,000 cells/well. Cells were maintained overnight at 37°C and 5% CO_2_ before use in experiments.

HNEpCs and J774.2 cells were then washed with sterile PBS (3 x 500 μL) and incubated in serum-free medium or GM-0111 (0, 30, 100, or 300 μg/mL) diluted in serum-free medium, for 1 h (37°C, 5% CO_2_). LL-37 (10 μM), or the LL-37 diluent only (controls), was then added to each well for 15 min. Supernatant (120 μL) was then collected, centrifuged, and subjected to ATP quantification under sterile conditions using an ENLITEN^®^ATP Assay System kit (Promega; Madison, WI) following the manufacturer’s instructions, and analyzed with a Tecan Infinite^®^200 PRO plate reader (Männedorf, Switzerland) in luminescence mode.

Fifteen minutes after the addition of LL-37 (10 μM), cells were then detached using Accutase and added to the remaining volume of their respective supernatant, and centrifuged. Cells were washed with PBS, centrifuged, and resuspended in 100 μL of PBS containing FITC-Annexin V (BioLegend; San Diego, CA) and 7-AAD (BioLegend; San Diego, CA) (10:2:1 PBS/FITC Annexin V/7-AAD) for 30 min at 37°C. The reaction was quenched with PBS. The cells were then centrifuged, resuspended in PBS, and analyzed using a Guava EasyCyte HT8 (Millipore; Billerica, MA) flow cytometer. These assays were performed in quadruplicate for each condition (n = 4).

### Morphologic change imaging of HNEpCs and J774.2 cells

HNEpCs and J774.2 cells were plated in μ-Slide 8 well glass bottom plates (Ibidi USA, Inc., Fitchburg, WI) and mLexamined for cell morphological changes under a Nikon TMS T009 (Nikon Inc., Melville, NY) microscope at 40x magnification before (time 0) and 60 min after the addition of vehicle (saline), LL-37 (10 μM), or GM-0111 (100 μg/mL) + LL-37 (10 μM).

### Cytokine quantitation by ELISA of HNEpCs

ELISA MAX^™^ Deluxe Sets were used for quantification of Human IL-6 and IL-8 protein (BioLegend; Sand Diego, CA). The ELISA plates were prepared the day before the assay per manufacturer instructions by addition of 100 μL of the appropriate Capture Antibody solution to each well, then sealing and incubating the plate overnight at 4°C. HNEpCs were plated at a density of 10,000 cells/well in 96-well plates and grown overnight (37°C, 5% CO_2_). The cells were then washed with sterile PBS (3 x 500 μL) and incubated in GM-0111 (30, 100, 300, or 1,000 μg/mL) or serum-free medium alone for 1 h (37°C, 5% CO_2_). Next, LL-37 (60 or 30 μM) or medium (controls) was delivered to each well. All conditions were performed in quadruplicate (n = 4). After 1 h incubation with LL-37, the supernatant from each sample was gently aspirated and analyzed by enzyme-linked immunosorbent assay (ELISA) according to manufacturer instructions. Absorbance was measured at 450 and 570 nm (570 nm subtracted) using a Tecan Infinite^®^200 PRO plate reader (Austria). A standard curve relating sample absorbance at 450 nm to concentration (pg/mL) of IL-6 or -8 was calculated with strong fit (r = 0.999) based on diluted standards of known concentration and used to calculate concentration of the samples.

### Caspase activation assay of HNEpCs and J774.2 cells

HNEpCs and J774.2 cells were plated and treated as described for death assays with GM-0111 (0, 1, 10, or 100 μg/mL) added in serum-free medium for 1 h (37°C, 5% CO_2_), followed by LL-37 (0 or 10 μM) for 15 min. FAM-FLICA^™^ assays (ImmunoChemistry Technologies; Bloomington, MN) specific to the detection of caspase-1, -3/7, and -8 activation were performed according to the manufacturer’s instructions and analyzed using a Guava EasyCyte HT8 flow cytometer (FITC filter). Cells were simultaneously labeled with 7-AAD (near red filter) as an indicator of terminal cell death. Cells with co-labeling of both active caspase and 7-AAD were selected for comparison between treatment groups. All conditions were performed in triplicate to quintuplicate (n = 3 to 5).

### Statistics

A normal Gaussian distribution was assumed to satisfy the requirements of the parametric one-way ANOVA test. However, formal nests of normality (Shapiro-Wilk and the D'Agostino & Pearson omnibus normality tests) could not be performed due to the small sample size per treatment group (n = 3 or 4). Pair-wise comparisons were made by one-way ANOVA, followed by Tukey’s post-test to adjust for multiple comparisons (P-value ≤ 0.05 indicates a statistically significant difference). For dose response curves a post-test for linear trend was also applied to the one-way ANOVA to determine significance of the apparent dose-response relationship. For the purpose of graphical representation and statistical analysis, ELISA data were transformed by addition of the absolute value of the most negative value in the data set (-3.0474 for IL-6 and -3.0727 for IL-8) as negative values of low or no signal groups resulted from prediction based on the curve of fit, though are physically nonsensical. Statistical tests and graphing were performed using Prism 6 for Windows (GraphPad Software; La Jolla, CA).

## Results

### LL-37 causes increased ATP secretion from HNEpCs and J774.2 cells, which is dose-dependently blocked by GM-0111

**Fig 1:**
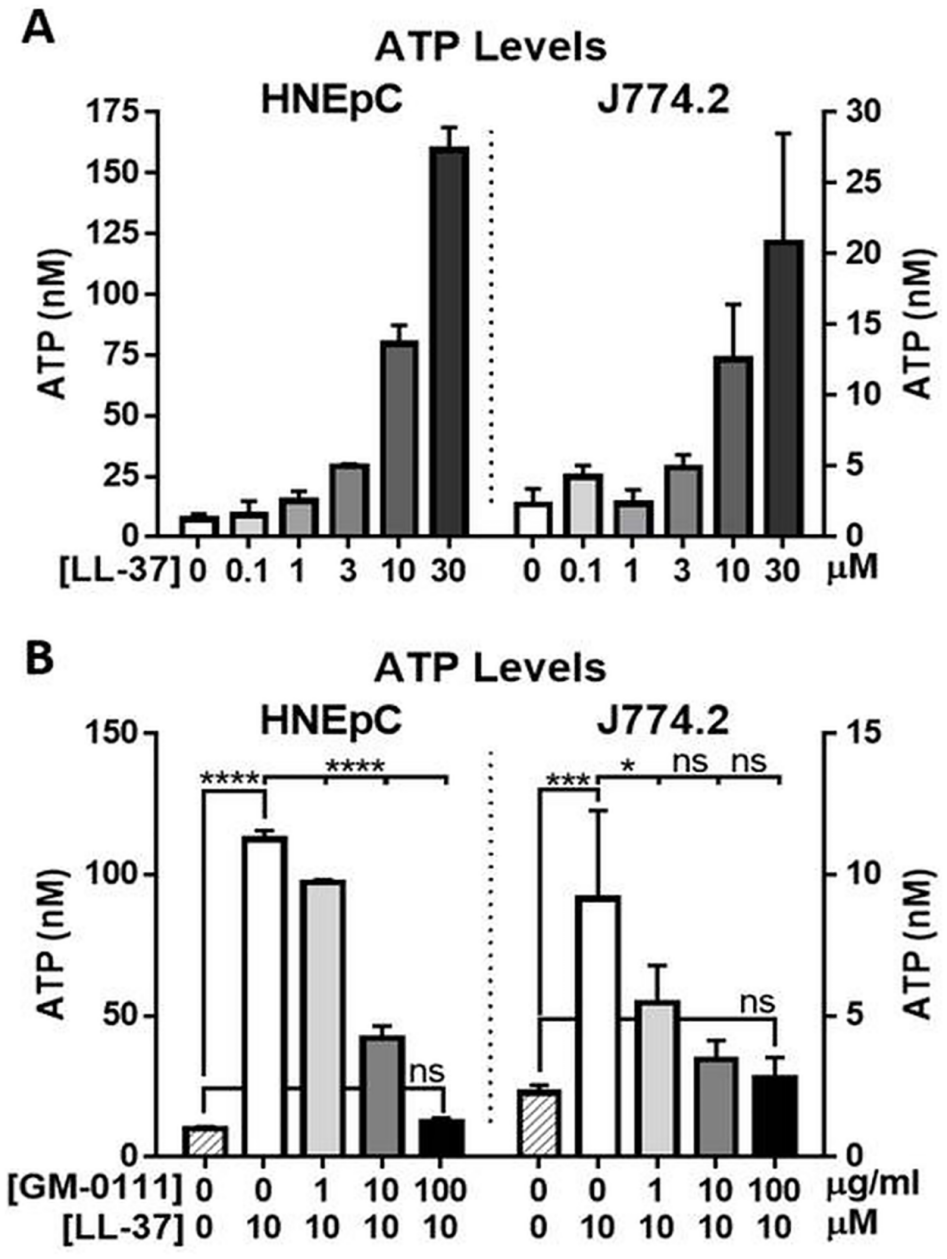
LL-37 causes a dose-dependent increase in ATP release from both HNEpC and J774.2 cells (A), which is blocked by GM-0111 in a dose-dependent manner (B). Each column represents the mean ± SD (n = 4). ****P ≤ 0.0001, ***P ≤ 0.001, *P ≤ 0.05, ns (not significant) *P* > 0.05.

The release of ATP from HNEpCs and J774.2 cells was measured in response to treatment with LL-37 ranging from 0 to 30 μM for 15 min. LL-37 caused a dose-dependent increase in ATP release from both HNEpCs and J774.2 cells (Fig 1A). A dose of 10 μM LL-37 was chosen for subsequent experiments, as this dose resulted in a pronounced cytotoxic effect while also maintaining a viable population (approximate median effective dose (ED_50_).

HNEpCs and J774.2 cells were then pre-treated with GM-0111 (0, 1, 10, or 100 μg/mL), followed by incubation with 10 μM LL-37. GM-0111 was found to significantly reduce LL-37-induced ATP release compared to cells treated only with LL-37 (Fig 1B) in a dose-dependent manner with a significant linear trend for this effect for both HNEpCs (*P* < 0.0001) and J774.2 cells (*P* < 0.001). For HNEpCs, mean ATP levels were increased from 9.8 nM ± 0.94 with no treatment to 112.6 nM ± 2.96 with 10 μM LL-37 (*P* < 0.0001), and levels were decreased to 12.44 ± 1.43 when pre-treated with 100 μg/mL GM-0111 in addition to the 10 μM LL-37 (*P* < 0.0001 for comparison to LL-37 alone). For J774.2 cells, mean ATP levels were increased from 2.27 nM ± 0.28 with no treatment to 9.15 nM ± 3.13 with 10 μM LL-37 (*P* < 0.001), and levels were decreased to 2.79 ± 0.72 when pre-treated with 100 μg/mL GM-0111 (*P* < 0.001 for comparison to LL-37 alone).

### LL-37 causes increased IL-6 and IL-8 release from HNEpCs, which is dose-dependently blocked by GM-0111

**Fig 2:**
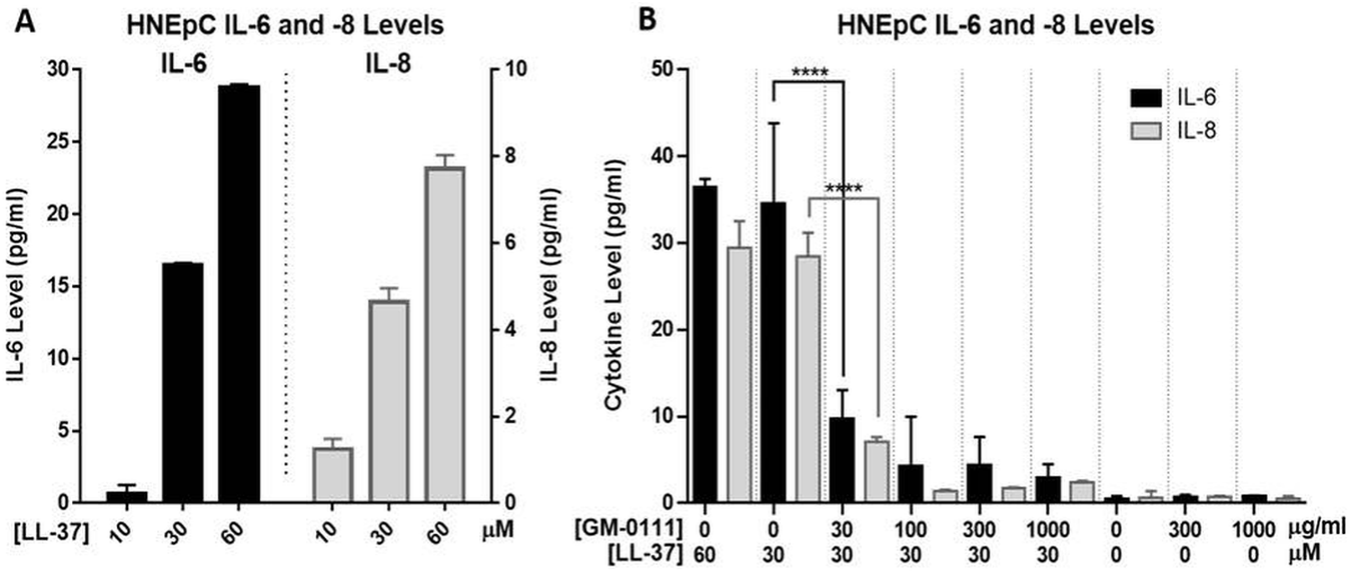
**Treatment of HNEpCs with LL-37 for 60 min results in a dose-dependent increase in cytokines IL-6 and -8 (A)**. Pre-treatment with GM-0111 causes significant dose-dependent reductions in LL-37-induced cytokine release (**B**). There is no significant effect of GM-0111 alone on IL-6 or -8 levels. ****P ≤ 0.0001.

HNEpCs were incubated with a dose ranging from 0 to 60 μM LL-37 for 15 or 60 min to determine the response of IL-6 and IL-8 cytokine levels. A robust increase in levels of both cytokines was seen at higher doses, but remained below the limit of detection at ≤ 3 μM (Fig 2A). Similar cytokine levels were observed with both 15 and 60 min treatment durations; 60 min incubation was chosen for subsequent experiments. A dose-dependent effect of LL-37 concentration on IL-6 and IL-8 levels, with a significant linear trend (*P* < 0.0001), was observed over the dose range of 10, 30, and 60 μM.

We then determined the effect of GM-0111 treatment on LL-37-induced IL-6 and IL-8 release from HNEpCs. Cells were pre-treated with GM-0111 (0, 30, 100, 300, or 1,000 μg/mL), then treated with 30 μM LL-37. This higher dose (relative to ATP experiments) was chosen for these experiments based on preliminary dose response curves demonstrating only a modest increase in cytokine levels with 10 μM. GM-0111 pre-treatment resulted in a dose-dependent decrease in LL-37-induced IL-6 and IL-8 levels (Fig 2B), with a significant linear trend (*P* < 0.0001). No significant effect on the levels of these cytokines was observed with GM-0111 treatment alone up to 1,000 μg/mL. Mean levels of IL-6 and IL-8, respectively, were increased from 0.47 pg/mL ± 0.33 and 0.61 pg/mL ± 0.80 in controls, to 34.59 pg/mL ± 9.21 and 28.47 pg/mL ± 2.72 with 30 μM LL-37 (*P* < 0.0001). When pre-treated with 100 μg/mL GM-0111 followed by 30 μM LL-37, cytokine levels were respectively decreased to 4.27 pg/mL ± 5.69 and 1.41 pg/mL ± 0.14 (*P* < 0.001 for comparison to LL-37 alone).

### LL-37 causes gross morphologic changes in HNEpCs and J774.2 cells, which are prevented with GM-0111

**Fig 3:**
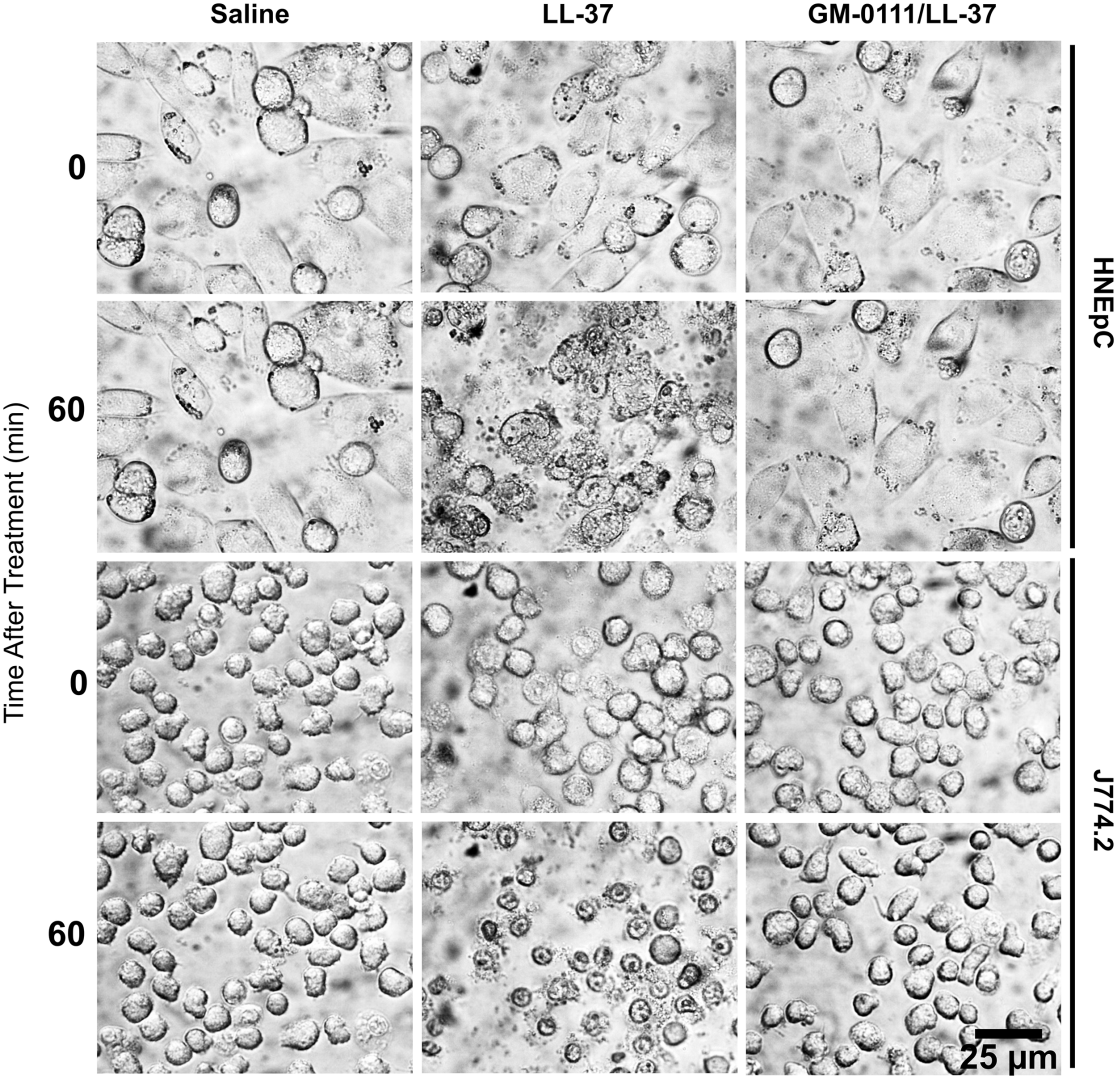
LL-37 induces morphologic change in HNEpCs (upper two rows) and J774.2 cells (bottom two rows), which are prevented with GM-0111 treatment. All images are at 40x magnification.

The effect of LL-37 and GM-0111 pre-treatment on cell death was assayed based on morphologic characteristics. Using light microscopy we obtained still images (Fig 3) following treatment of HNEpCs and J774.2 cells with either phosphate buffered saline (PBS; vehicle control), LL-37 (10 μM), or both GM-0111 (100 μg/mL) and LL-37 (10 μM). Representative images are shown at time points of 0 and 60 min after treatment (Fig 3). When treated with LL-37, gross morphologic changes consistent with cell death were observed, including cell swelling and rupture over 15 min (Fig 3, column 2). When pre-treated with GM-0111 (Fig 3, column 3), these morphologic changes were not observed; the cells were grossly indistinguishable from controls.

### LL-37 causes increased death of HNEpCs and J774.2 cells, which is dose-dependently blocked by GM-0111

**Fig 4:**
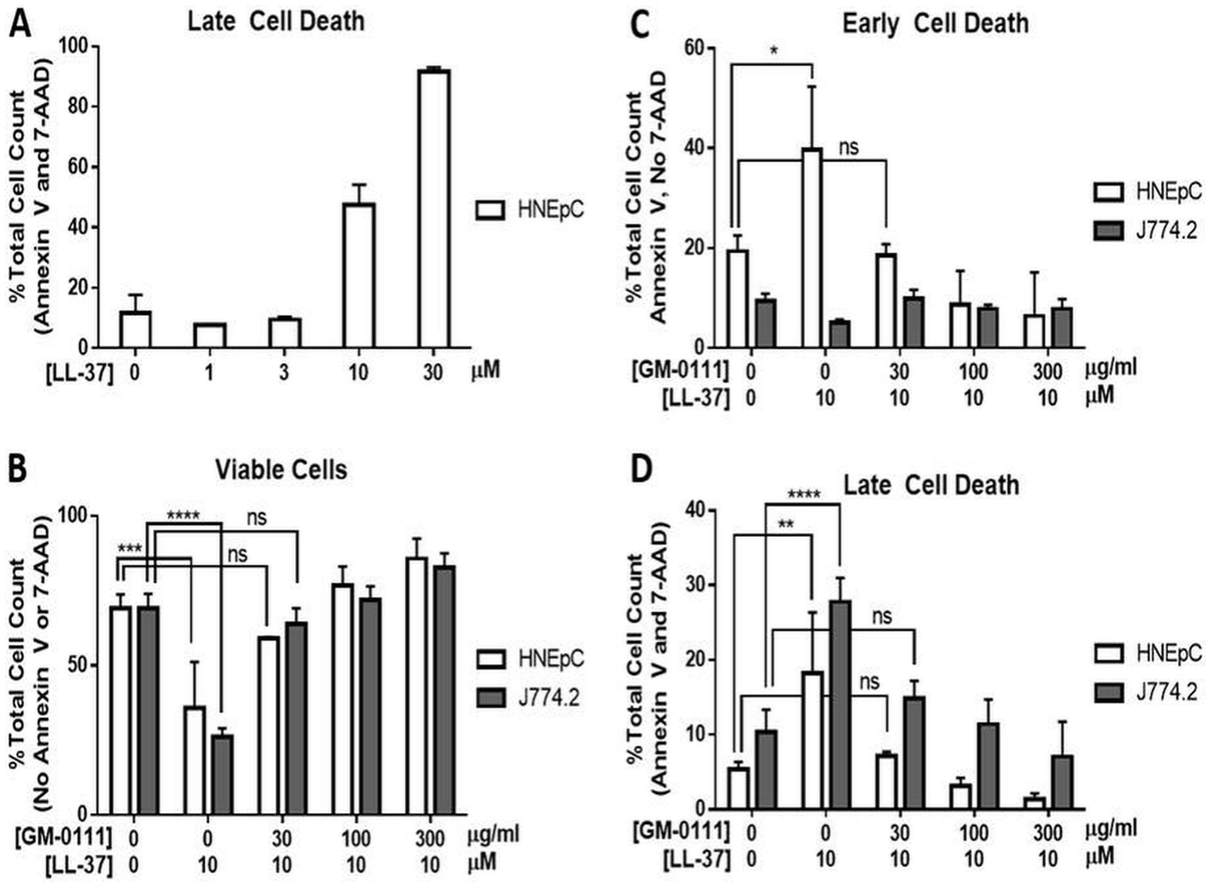
LL-37 increases cell death, which is reduced by GM-0111. **A**. Treatment with LL-37 alone results in a dose-dependent increase in late cell death (Annexin V^+^/7-AAD^+^) in HNEpCs. **B**. The percentage of total HNEpCs and J774.2 cells that are viable (Annexin V^-^/7-AAD^-^) is significantly reduced with LL-37 treatment, and this is dose-dependently reversed with GM-0111. **C**. The percentage of cells undergoing early death (Annexin V^+^/7-AAD^-^) is significantly increased in HNEpCs treated with LL-37, and the effect is reversed with GM-0111. **D**. The percentage of HNEpCs and J774.2 in late cell death (Annexin V^+^/7-AAD^+^) is also significantly increased with LL-37 treatment and dose-dependently reversed with GM-0111. ****P ≤ 0.0001, ***P ≤ 0.001, **P ≤ 0.01, *P ≤ 0.05, ns (not significant) *P* > 0.05. The data represent the means ± SD (n = 4).

Cell death was quantitated by flow cytometric detection of respective early and late cell death markers, Annexin V and 7-aminoactinomycin D (7-AAD).[34,35] HNEpCs and J774.2 cells were treated first with a range of LL-37 doses for 15 min, to determine a dose that induced pronounced cytotoxic effect while maintaining a viable population. The data demonstrated a clear dose-dependent increase in the number of Annexin V- and 7-AAD-postive cells, with near complete cell death at 30 μM, and approximately 55% (approximate ED_50_) of the total Annexin V- and 7-AAD-positive cell population when treated with 10 μM LL-37 for 15 min (Fig 4A, HNEpCs). These findings were consistent with our previous data; and 10 μM was used for subsequent studies.

HNEpCs and J774.2 cells were pre-treated with GM-0111 (0, 30, 100, or 300 μg/mL), followed by incubation with 10 μM LL-37, labeled for Annexin V and 7-AAD, and then subjected to flow cytometry. GM-0111 reduced LL-37-induced cell death in a dose-dependent manner when compared to cells treated only with LL-37 (Fig 4B–4D). The percentage of cells negative for both Annexin V and 7-AAD (considered viable) was significantly reduced from 68.96% ± 4.72 and 69.04% ± 4.89 with no treatment, to 35.91% ± 15.30 (*P* < 0.001) and 26.04% ± 3.02 (*P* < 0.0001) with 10 μM LL-37, for HNEpC and J774.2 cells, respectively (Fig 4B). This was reversed in a dose-dependent manner with GM-0111 pre-treatment (*P* < 0.0001; linear trend). Interestingly, at the highest dose of GM-0111 pre-treatment (300 μg/mL GM-0111 with 10 μM LL-37), cellular viability exceeded even that of the controls, indicating that GM-0111 may possess cytoprotective effects; 68.96% ± 4.72 and 69.04% ± 4.89 viable controls compared to 85.68% ± 6.73 and 82.65% ± 4.83 (*P* < 0.01) for HNEpC and J774.2 cells, respectively, though this did not reach statistical significance for HNEpCs.

The percentage of cells demonstrating late cell death changes (Annexin V and 7-AAD positive) increased significantly after treatment with 10 μM LL-37 in HNEpCs (*P* < 0.01) and J774.2s (*P* < 0.0001); a dose-dependent decrease in late cell death occurred with GM-0111 pre-treatment (Fig 4D) (*P* < 0.0001; linear trend). In HNEpCs, early death (Annexin V-positive, 7-AAD-negative) followed the same trend as observed with late cell death; there was a significant increase in the percent of cells with early cell death changes after treatment with 10 μM LL-37 (*P* < 0.05) and a dose-dependent decrease with GM-0111 pre-treatment (*P* < 0.0001; linear trend). However, in the J774.2 cells there was a significant decrease in early cell death with 10 μM LL-37 (*P* < 0.01) which was reversed with GM-0111 pre-treatment (*P* < 0.01 for 10 μM LL-37 alone compared to pre-treatment of 10 μM LL-37 followed by 30 μg/mL GM-0111); there was no apparent dose-dependent relationship of GM-0111 and the percentage of cells in early cell death as the percentage remained insignificantly different from controls at doses of 30 μg/mL GM-0111 and higher (Fig 4C).

### LL-37 induces caspase-1 and -8 activation in HNEpCs and JJ74.2 cells, and GM-0111 protects against activation

**Fig 5:**
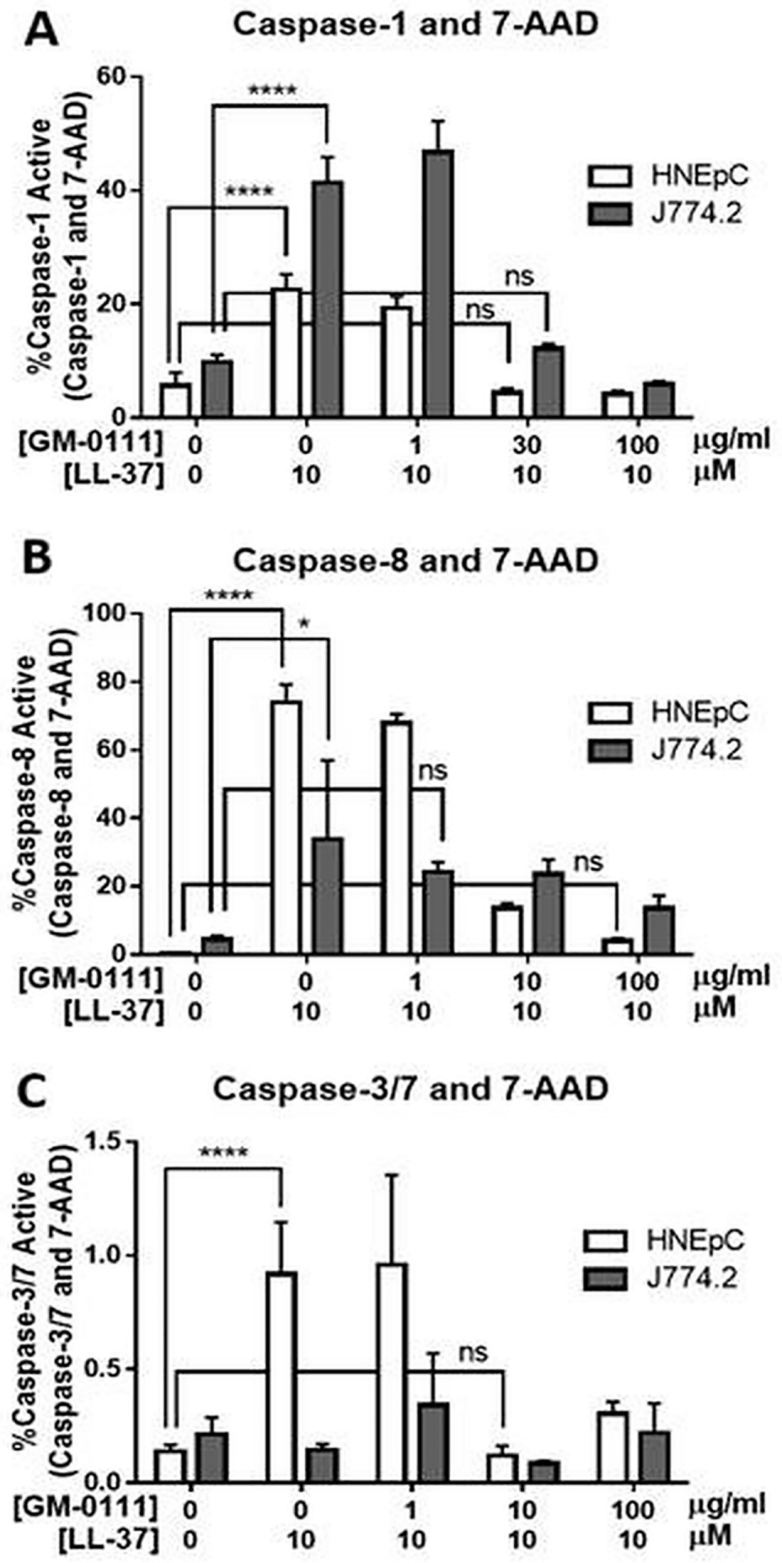
In cells committed to terminal cell death, caspase-1 and -8 activity is increased with LL-37 treatment and reduced with GM-0111. **A**. The percentage of total HNEpCs and J774.2 cells with active caspase-1 and 7-AAD (late cell death marker) is significantly increased with LL-37 treatment, and this is dose-dependently reversed with GM-0111 treatment. **B**. The percentage of total HNEpCs and J774.2 cells with active caspase-8 and 7-AAD is significantly increased with LL-37 treatment and dose-dependently decreased with GM-0111. ****P ≤ 0.0001, *P ≤ 0.05, ns (not significant) P > 0.05. The data represent the means ± SD (n = 2–5). Note the differences in y-axis scale (panel B y-axis maximum only 1.5%), adjusted to allow visual resolution but heights of bars are not comparable between panels. **C**. There is a statistically significant though minimal (<1%) increase in caspase-3 or -7 activity in HNEpCs also positive for 7-AAD after LL-37 treatment, and the effect is reversed with GM-0111. There is no significant change in caspase-3 or -7 activity with LL-37 or GM-0111 in J774.2 cells.

Caspases are enzymes that have unique roles in regulating the phenotype of cellular death.[36] After J774.2 cells were incubated with 10 μM LL-37, the percentage of total cells that were both dead (7-AAD positive) and containing active caspase-1 increased from 5.72% ± 2.24 to 22.51% ± 2.76 (*P* < 0.0001) in HNEpCs, and from 9.82% ± 1.27 to 41.35% ± 4.53 (*P* < 0.0001). For both cell types, caspase-1 activation was inhibited dose-dependently by GM-0111 pre-treatment (*P* < 0.0001; linear trend), with the percentage of cells both dead and expressing active caspase-1 reduced to similar levels as controls when treated with both 10 μM LL-37 and 30 μg/mL GM-0111 (Fig 5A).

The percentage of the total cells that were both dead and expressing active caspase-8 increased significantly with 10 μM LL-37 treatment, from 0.42% ± 0.09 to 73.95% ± 5.25 (*P* < 0.0001) in HNEpCs, and from 4.38% ± 1.19 to 33.77% ± 23.19 (*P* < 0.05) in J774.2 cells. Caspase-8 activation was dose-dependently blocked by GM-0111 pre-treatment for both cell types (HNEpC: *P* < 0.0001; J744.2: *P* < 0.05; linear trends) (Fig 5B).

### LL-37 does not induce caspase-3 or -7 activation in HNEpCs or J774.2 cells

For J774.2 cells, no significant effect of LL-37 treatment on the percentage of total cells that were 7-AAD positive and demonstrated caspase-3 or -7 activity was observed, despite indications of cell death and toxicity (*i*.*e*., morphologic changes, ATP release, and Annexin V/7-AAD labeling). For HNEpCs, a statistically significant, but biologically negligible increase in 7-AAD/caspase-3 and -7-positive cells was observed (0.14% ± 0.03 to 0.92% ± 0.23) after 10 μM LL-37 treatment. This small increase in caspase-3 and -7 activity decreased in a dose-dependent fashion with GM-0111 pre-treatment (*P* < 0.0001; linear trend).

## Discussion

The pathogenesis of RS-associated sinonasal mucosal inflammation is complex and poorly understood despite extensive research into its pathophysiology. The heterogeneity of the disease has made it challenging to determine specific molecular mechanisms and inflammatory pathways resulting in the end phenotype of sinonasal inflammation, as well as to develop new therapies to prevent and treat this condition. The objectives of this work were to determine (1) the molecular mechanisms by which the inflammatory-modulating peptide LL-37 might contribute to sinonasal mucosal inflammation and cell death, and (2) how a non-steroidal, synthetic GAG (GM-0111) prevents this.

Our data demonstrate, that LL-37 treatment results in pro-inflammatory signaling, with increased levels of ATP, IL-6, and IL-8, and that these effects are dose-dependently blocked by GM-0111. In response to stress and damage, cells secrete ATP, stimulating pro-inflammatory mediators through purinergic receptor activation.[37] Increased ATP release from human intestinal epithelial cells and human urothelial cells has been linked to cell death within the context of inflammatory gut and bladder disease, respectively.[35,38] Similarly, increased levels of the pro-inflammatory cytokines IL-6 and IL-8 have been demonstrated from airway cells after stimulation by bacteria,[39] viral infection,[40] and in nasal epithelial cells after challenge with an antigen.[41]

Significantly increased levels of IL-6 have also been demonstrated in patients with CRS compared to controls,[42,43,44,45,46,47] and IL-8 levels have been previously shown to correlate with nasal symptoms in acute respiratory infections and in patients with CRS.[48,49] IL-8 is also increased in the sinonasal mucosal tissue and nasal discharge of patients with CRS relative to controls [47,50] and positively correlated with radiographic evidence of CRS-specific disease severity as well.[51] Taken together, our findings are consistent with what has been previously identified in CRS.

Our data show that LL-37 induces marked cellular morphology changes that are consistent with cell death and that GM-0111 protects against these changes. Necrosis is morphologically distinct from programmed cell death (apoptosis). Necrosis is characterized by cytoplasmic swelling (oncosis), moderate chromatin condensation, and rupture of the plasma membrane; in contrast to the cell rounding, nuclear fragmentation, and blebbing of the plasma membrane that leads to phagocytes of apoptotic bodies.[52,53] Observation of both HNEpCs and J774.2 cells after treatment with LL-37 revealed morphologic changes of cellular membrane rupture and extrusion of intracellular contents, but also cellular swelling as seen with the process of oncosis (Fig 3). These morphologic changes of cell death were prevented with GM-0111. These particular morphologic findings appear to be consistent with the cell death processes of necrosis and/or pyroptosis, within the context of our other enzymological data. Pyroptosis has been described morphologically as cell rupture, followed by membrane “re-sealing,” and cell swelling with nuclear condensation.[54]

Similarly, cells externalize phosphatidylserine (PS) on an intact cell membrane during oncosis in early necrosis, as well as when undergoing pyroptosis.[55,56,57,58] Pyroptotic cells also undergo PS-dependent phagocytosis.[58] Observed changes in the percentage of cells positive for Annexin V without 7-AAD (Fig 4C) therefore are interpreted to indicate early cell death without inferring a specific cell death process, as Annexin V binds PS when exposed by externalization on an intact membrane such as in early apoptosis or after loss of cell membrane integrity. HNEpCs were found to have a significant increase in early cell death with LL-37 that was abolished with GM-0111, whereas J774.2 cells did not demonstrate significant changes with treatment, demonstrating a difference in the time course of cell death. Interestingly, a higher dose of GM-0111 reduced cell death below that observed with controls, suggesting a mechanism of cytoprotection beyond simple sequestration of LL-37 by charge neutralization, which would be expected to prevent the adverse effects of LL-37 without impact on basal levels of viability.

Examining the specific death pathways activated by LL-37 and blocked by GM-0111, we assayed which caspases were involved. We observed that LL-37 caused robust increases in caspase-1 and -8, but not in caspases-3/7 in HNEpCs and J774.2 cells. All caspase activity was dose-dependently blocked with GM-0111 pre-treatment. Caspase-3, -7, and -8 serve roles in apoptotic cell death, whereas Caspase-1 has a distinct role in pro-inflammatory cell death.[59,60] Caspase-3 is specifically required for characteristic features of apoptotic cell death such as DNA fragmentation.[61,62,63] Here, caspase activity was measured only in cells also positive for 7-AAD for selection of cells terminally committed to death. These data suggest that cell death is not occurring through classical apoptosis.

Caspase-1 is associated with the pro-inflammatory death mechanism of pyroptosis,[64] as demonstrated in macrophages and dendritic cells.[65,66] This cell death pathway is initiated when Nod-like receptors (NLRs) respond to a variety of cellular danger signals, including extracellular ATP, forming the multiprotein inflammasome complex. The inflammasome activates caspase-1, which in turn initiates pyroptotic cell death and pro-inflammatory cytokines.[66,67] Increased caspase-1 activity in response to LL-37 is therefore consistent with a pro-inflammatory model of pyroptotic cell death and increased inflammatory signaling, as opposed to an anti-inflammatory apoptotic cell death mediated by caspase-3 and -7.

**Fig 6:**
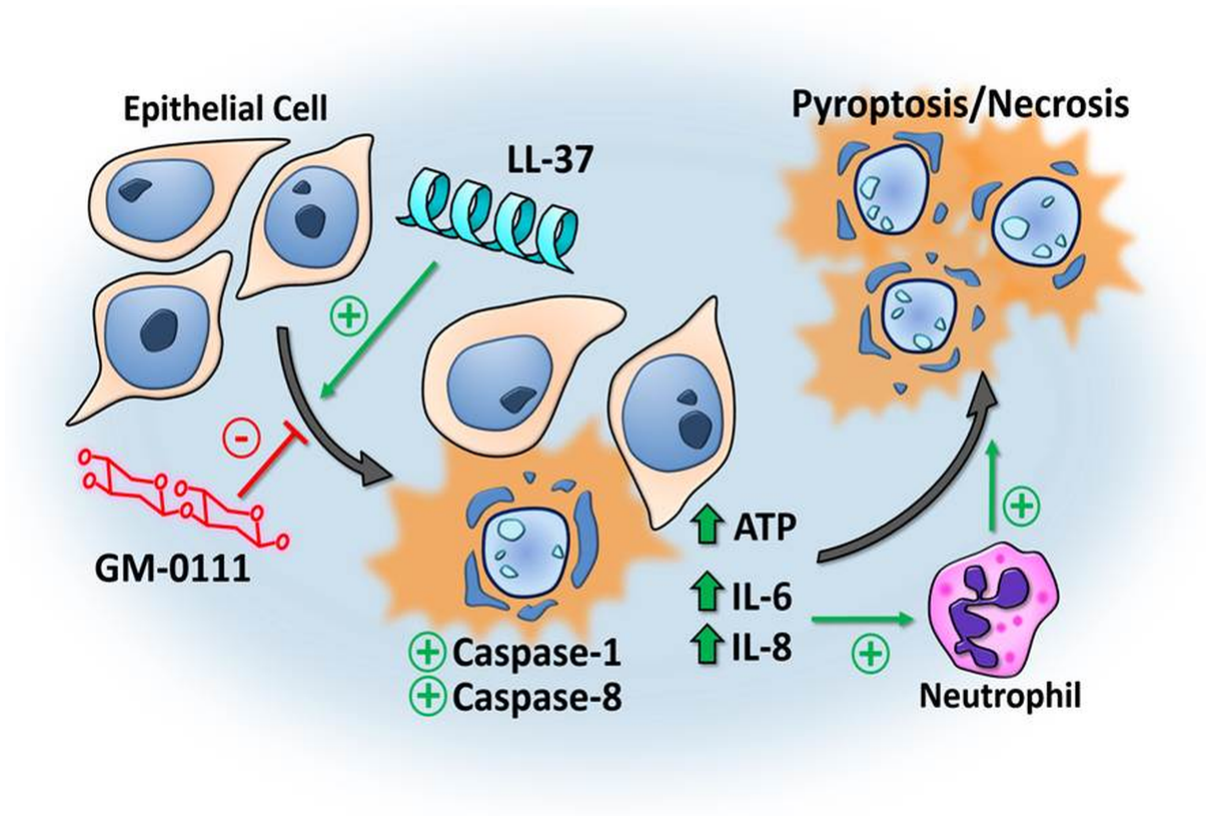
Diagram of the proposed mechanism of LL-37-induced cell death and protection from GM-0111. Nasal epithelial cells subjected to LL-37 demonstrate a pro-inflammatory response, characterized by increased ATP, IL-6, and -8 production and pyroptosis and/or necrosis via caspase-1 and -8 but not caspase-3 or -7 activation. These changes are prevented by GM-0111 treatment. IL-6 and -8 promote an inflammatory response *in vivo* through the recruitment of neutrophils and further inflammatory signaling in a positive feedback loop. The process of pro-inflammatory cell death is propagated to nearby cells due to these changes in the local environment, resulting in an unchecked cytotoxic response initiated by LL-37.

While active caspase-8 is classically known to activate caspase-3 in apoptotic cell death,[64] there is evidence for more diverse roles of caspase-8 in inflammatory signaling and caspase-1-mediated cell death by pyroptosis.[68,69,70,71] Our findings thus support a mechanism of LL-37-induced pyroptotic cell death in HNEpCs and J774.2 cells, though observed rapid morphologic changes also suggest the possibility of some necrotic/non-programmed cell death (Fig 6).

A limitation to this study is the difficulty in validating LL-37-induced inflammation as an accurate *in vitro* model of RS-associated sinonasal mucosal inflammation. Reported endogenous levels of LL-37 within the context of airway inflammation range from 2 to 8 nM.[18,72,73] In this study, the supraphysiological concentrations of LL-37 employed fell within the range of previous *in vitro* functional studies using LL-37 to induce different biological outcomes [74,75,76] and were optimized to produce a robust response that could be reproducibly measured. Findings from the present study and previous work support the plausibility of this model by similarities to clinical findings of patients with RS. Our prior animal model demonstrated histologic changes of inflammation and a similar inflammatory infiltrate and cell death to that seen in RS-associated sinonasal disease.[26] Molecular changes from the present study are more difficult to relate to *in vivo* findings from patients, due to the heterogeneity of the disease process and likely multiple mechanisms that may result in a similar disease phenotype. Perpetual pro-inflammatory processes initiated by the inflammasome and caspase-1, as well as the downstream pro-inflammatory effects of pyroptotic and/or necrotic cell death is consistent with the seemingly uncontrolled sinonasal inflammation observed in RS.

In summary, our findings suggest that LL-37-induced cell death of HNEpCs and J774.2 cells occurs via the mechanism of pyroptosis or a combination of pyroptosis/necrosis. We demonstrate that a synthetic GAG (GM-0111) dose-dependently prevents increased inflammatory mediator production and apparent pyroptotic and/or necrotic cell death induced by LL-37, supporting its potential utility as a novel therapeutic for upper airway inflammatory conditions. Further work is needed to assess the effects of other therapeutics in this inflammatory model, as well as to apply GM-0111 in other models and to further validate its utility in different contexts of sinonasal mucosal inflammation (*e*.*g*., allergic and/or infectious *in vivo* models).
